# Management Challenges and Potential Malabsorption of Valproic Acid in a Patient with Bipolar Disorder and Gastrointestinal History

**DOI:** 10.1155/2024/1426930

**Published:** 2024-07-25

**Authors:** James Kwok, Janeline Wong, Kye Kim

**Affiliations:** ^1^ Virginia Tech Carilion School of Medicine, Roanoke, VA, USA; ^2^ Carilion Clinic, Roanoke, VA, USA

## Abstract

Bipolar disorder is a chronic psychiatric condition typically managed using mood stabilizers such as valproic acid, lithium, and atypical antipsychotics, the former which is absorbed in the gastrointestinal tract. This case report presents the challenges encountered in managing bipolar disorder in a patient with a history of extensive gastrointestinal (GI) issues. The patient was initially treated with lithium but experienced adverse effects, prompting a switch to valproic acid (VPA) tablets. However, due to ongoing GI problems unrelated to her medication and to help improve tolerability, the patient underwent multiple medication formulation changes, including Depakote delayed release tablets, Depakene liquid, and Depakote sprinkle capsules. However, the patient's VPA levels decreased below therapeutic levels after the formulation changes despite medication compliance. This case highlights the importance of considering GI issues in optimization of a treatment plan for patients with bipolar disorder.

## 1. Introduction

Bipolar disorder is a chronic psychiatric condition often managed using mood stabilizers such as valproic acid and lithium. However, in patients with gastrointestinal (GI) history, the absorption and bioavailability of medications can be affected, posing challenges in treatment effectiveness.

The mechanism of action of valproic acid (VPA) in neuropsychiatric diseases is not fully understood [1]. However, evidence shows that VPA inhibits gamma-aminobutyric acid (GABA) transaminase, a degradation enzyme, leading to an increase in the inhibitory neurotransmitter GABA. This in turn results in an overall decrease in neuronal excitability [2]. Although the primary FDA indication for VPA is as therapy for seizures, off-label usage of VPA includes maintenance therapy for bipolar disorder and treatment for acute bipolar depression [3].

Pharmacokinetics of the different formulations of VPA have been well studied. VPA is metabolized in the liver via the glucuronidation pathway and then is eventually excreted in the urine. As a weak inhibitor of cytochrome P450 (CYP) enzymes, VPA has multiple drug interactions that necessitate close monitoring and possible dose adjustments when administered with CYP inducers, e.g., carbamazepine and phenytoin [4].

As VPA is readily absorbed from the GI tract, oral administration of VPA can be influenced by ingestion of food as well as other factors that affect GI absorption. Typical bioavailability of VPA in most formulations is 90%–100%. Additionally, VPA has a high affinity for plasma proteins, especially albumin, which allows it to rapidly distribute to the central nervous system via fatty acid transport systems among other mechanisms [5].

Because of its potential interactions and variable efficacy, serum VPA levels are monitored regularly. Therapeutic ranges are set at 50–100 mcg/mL in epilepsy and 50–125 mcg/mL in mania [5]. These serum levels, along with clinical presentation of the patient, can provide evidence for therapeutic efficacy of VPA as well as optimization of medication administration.

VPA has multiple formulations and methods of administration. Depakote (divalproex sodium), a form of VPA with sodium valproate, comes in oral tablets that can be given in an extended release or delayed release form. Extended release is designed to be taken once daily by releasing VPA over 24 hr, whereas delayed release is coated to prevent early GI dissolution. Delayed release should be taken twice a day. There is also a capsule sprinkle form for those with difficulty swallowing. Depakene is a liquid VPA that can be administered in capsule form or as a syrup [6]. Additionally, IV VPA can be administered to the patient. Factors affecting selection of VPA formulation include patient comfort, adverse effects, and therapeutic efficacy.

## 2. Case Presentation

The patient is a 65-year-old, retired Caucasian female, with a 15-year history of bipolar disorder. She also had an extensive GI history that included a gastric bypass, gastroesophageal reflux disease, dysphagia, delayed gastric emptying, exploratory laparotomy, and a foreign body removal procedure. Other medical conditions include hypertension, osteoarthritis, mild hearing difficulty, and bilateral cataracts. She is presently on lisinopril, omeprazole, and meloxicam for those medical conditions. Additionally, she takes mirtazapine 7.5 mg at bedtime for insomnia. Her GI conditions preexisted a trial of valproic acid. Her bipolar disorder had previously been treated with lithium up until 3 years ago when she began developing tremors and the occasional fall. This prompted a switch to valproic acid (Depakote) tablets 500 mg delayed release twice daily (BID). Four days after initiation of Depakote, her valproic acid blood levels were 18.4 (therapeutic range: 50–125). Although her valproic acid levels were not at the therapeutic level yet, we were reassured that she was tolerating and absorbing her medication properly, as studies have shown that it takes up to 14 days for valproic acid to reach maximum concentration.

Over the next 2 years, the patient remained on the same formulation of Depakote tablets 500 mg delayed release BID. During this time, approximately 6 months after initiating Depakote treatment, another valproic acid blood level was taken that revealed a level of 82.2, suggesting therapeutic benefits. However, the patient reported frequent GI upset unrelated to Depakote, leading to a switch to Depakene liquid formulation (125 mg twice daily) for improved tolerability. Of note, during this period, the patient also faced many significant psychosocial stressors, including her husband's diagnosis of early-onset dementia.

Three days after starting Depakene, the patient's valproic acid blood level was measured at 60.1. We were not concerned as the patient was still at therapeutic levels and had just started on a new formulation that was lower than her original dose. Six months after switching to Depakene, however, the patient began reporting difficulty with the taste of Depakene and expressed feelings of being overwhelmed, perceiving her medication to be ineffective. Considering her dysphagia history and to enhance tolerability, we decided to switch the patient to Depakote sprinkle capsules 125 mg delayed release twice daily. The patient was educated on how to properly take her new medication. Again, however, 4 months later, the patient began reporting issues with tolerability, and she was transitioned back to the original Depakote tablets delayed release, but this time at a dose of 250 mg BID. Patient remained on this dose until her most recent visit.

During the patient's last visit ~4 months after switching back to the original Depakote tablets, she reported some impulsivity for the last couple months and had endorsed some trouble sleeping despite being on mirtazapine for sleep. Our plan was to increase her bedtime Depakote dose to 500 mg for additional sleep aid and for better impulsivity control. Before changing her medication, we decided to evaluate her valproic acid blood levels, which were found to be less than 3.2, far below the therapeutic range (50–100). The subsequent blood level continued to remain subtherapeutic. Review of pharmacy records and review of documentation of communication between the patient and her primary provider indicated that there was no reason to suspect medication noncompliance.

## 3. Discussion

This case report highlights several important considerations in the management of bipolar disorder in a patient with a complex GI history. In our case, the patient's GI history enabled us to suspect malabsorption, but to our knowledge, there is only one published case report of potential VPA malabsorption in a patient with GI comorbidities. The only other case report showing differing efficacies of valproic acid formulations was in a patient that did not have any specific GI conditions. Absorption and efficacy of VPA are highly dependent on formulation and on individual patient factors that may not be obvious to the prescriber.

There have been only one previous reported case reports of VPA malabsorption in a patient with GI issues. In 2004, a 68-year-old female with treatment-resistant bipolar depression and a past medical history of removal of small intestine and chronic diarrhea showed marked improvement when her valpromide (VPD), a derivative of VPA, was switched to VPA. As a prodrug of VPA, VPD is transformed to VPA in the intestine and then absorbed in the mucus membranes [7]. In a patient with a missing part of her intestine, therefore, the bioavailability of VPA is decreased when dosed as VPD. This case provides evidence for potential clinical differences in formulations of VPA, specifically VPA versus VPD, for the treatment of bipolar depression.

An example of malabsorption without a clear history of GI issues demonstrates the importance of individualized medicine. In 2017, a 30-year-old woman with a 17-year history of juvenile myoclonic epilepsy was given slow-release tablets of VPA after which low serum concentrations of the drug were observed. Due to low medication bioavailability, the patient had persistence of her seizure episodes. When the formulation was changed to syrup and enteric-coated tablets, a normal drug concentration was observed with a concurrent decrease in number of seizures and concurrent improvement of EEG recordings [8].

Additionally, with regard to our patient, her significant psychosocial stressors may have impacted her response to treatment. In particular, issues related to her husband's early-onset dementia diagnosis as well as her role as primary caretaker for her grandchild were sources of chronic stress. Severe chronic stress has been implicated as one of several factors that can alter an individual's gut microbiome [9], which in turn has been shown to affect the absorption and bioavailability of medications [10]. Our patient's numerous stressors certainly support the possibility that stress-induced alteration of her gut microbiome contributed to the malabsorption of VPA.

## 4. Conclusion

Most importantly, this case suggests that physicians should investigate possible sources of malabsorption with persistent subtherapeutic levels. Patients with a GI history may require closer monitoring of valproic acid levels. Further study on the efficacy of different valproic acid formulations for patients with a GI history is warranted. However, as demonstrated in the second case, even patients without a clear history of GI issues may present with decreased VPA efficacy. With the variety in formulations available for VPA, physicians can better optimize administration of VPA for bipolar disorder to reduce risks of side effects while maximizing clinical benefits.

## Data Availability

The case report data used to support the findings of this study are included within the article.
